# Pre-operative Spermatic Cord Ultrasonography Helps to Reduce the Incidence of Metachronous Inguinal Hernia in Boys

**DOI:** 10.3389/fped.2018.00156

**Published:** 2018-06-04

**Authors:** Shoujiang Huang, Xiuzhen Yang, Canping Li, Yunzhong Qian, Zhengyan Zhao, Jianfeng Liang

**Affiliations:** ^1^Ambulatory Surgery, Children's Hospital, ZheJiang University School of Medicine, Hangzhou, China; ^2^Department of Ultrasonography, Children's Hospital, ZheJiang University School of Medicine, Hangzhou, China; ^3^General Surgery, Children's Hospital, ZheJiang University School of Medicine, Hangzhou, China; ^4^Children's Hospital, ZheJiang University School of Medicine, Hangzhou, China

**Keywords:** pre-operative spermatic cord ultrasonography, metachronous inguinal hernia, contralateral inguinal hernia, inguinal hernia, herniorrhaphy

## Abstract

**Background/purpose:** Thickening of the spermatic cord is a clinical sign of an inguinal hernia. We therefore tested whether pre-operative spermatic cord ultrasonography could reduce the incidence of metachronous inguinal hernia (MIH).

**Methods:** Boys under 2 years old with an initial unilateral inguinal hernia were enrolled in this study. In whom the width of the asymptomatic-sided spermatic cord was ≥0.5 cm, these patients underwent contralateral groin exploration. Age at initial operation, weight, initial operation side, the sonographic width of the spermatic cord, the operative findings and presence of MIH were recorded, and the relationship among them was studied. Boys in the US group underwent an open herniorrhaphy with pre-operative ultrasound examination; the non-US group included boys who did not undergo a pre-operative ultrasound examination. A receiver operator curve (ROC) analysis was performed to evaluated predictive value of the sonographic width of the spermatic cord for contralateral hernia.

**Results:** A total of 24 months' follow-up data were obtained from 1,793 boys (US group 1,162, non-US group 631). In the US group, the width of the hernia-sided spermatic cord (0.75 ± 0.18 cm) was larger than the normal side (0.37 ± 0.05 cm, *P* < 0.001). And the width of normal side spermatic cord had no significant difference between the groups regarding other factors such as age and weight. In whom the width of the asymptomatic-sided spermatic cord was ≥0.5 cm, the corresponding incidence of CIH was 86.4% (57/66). The width of the spermatic cord predicted the presence of contralateral hernia with ROC area under the curve = 0.943 (95% CI = 0.919–0.966). The total incidence of MIH was 4.1% (74/1793). The incidence of MIH in the US group was 2.2% (25/1162) much lower than 7.8% (49/631) in the non-US group (*P* < 0.001). If the width of the asymptomatic-sided spermatic cord was 0.5 cm and 0.54 cm, the corresponding sensitivity was 0.682 and 0.294, respectively, the corresponding specificity was 0.991 and 1.000, respectively.

**Conclusion:** If the width of the asymptomatic-sided spermatic cord of boys with initial unilateral inguinal hernia sonographic width was ≥0.5 cm, contralateral groin exploration was recommended, and it help to reduce the incidence of MIH.

## Introduction

The incidence of metachronous inguinal hernia (MIH) after an initial unilateral herniorrhaphy was reported to be 5.2–12.3% (1–3). It is still controversial whether contralateral exploration is necessary in children with an initial unilateral inguinal hernia during conventional open herniorrhaphy. Laparoscopic hernia repair (LHR) can confirm the presence of contralateral patent processus vaginalis (CPPV). However, the incidence of CPPV evaluated by laparoscopy is 20–51.5%, which is much higher than the natural incidence of contralateral inguinal hernia (CIH) or MIH(4, 5). Ultrasonography is a safe and non-invasive method which helps to diagnose inguinal hernia and detect the presence of CPPV (6–8). According to these studies, an ultrasound scan can be used to examine the groin or internal ring to detect CPPV. Thickening of the spermatic cord is a clinical sign of inguinal hernia. Therefore, we tested whether spermatic cord ultrasonography could predict CIH to reduce the incidence of MIH.

## Methods

This retrospective study was approved by the Institutional Review Board of the Children's Hospital, Zhejiang University School of Medicine. From January 2010 to February 2014, 7558 boys with an inguinal hernia underwent open herniorrhaphy in 1 day care department (the predecessor of the department of ambulatory surgery) or the general surgery department from January 2014 to February 2014. Some of these patients received an ultrasound examination. Boys aged 1–2 years (13–24 months) who underwent open herniorrhaphy with pre-operative ultrasound examination comprised the US group, boys aged 1–2 years (13–24 months) without a pre-operative ultrasound examination comprised the non-US group. Boys with a clinical bilateral inguinal hernia or a bilateral inguinal hernia diagnosed by ultrasound examination were excluded from this study.

### Spermatic cord ultrasonography

**Figure 1:**
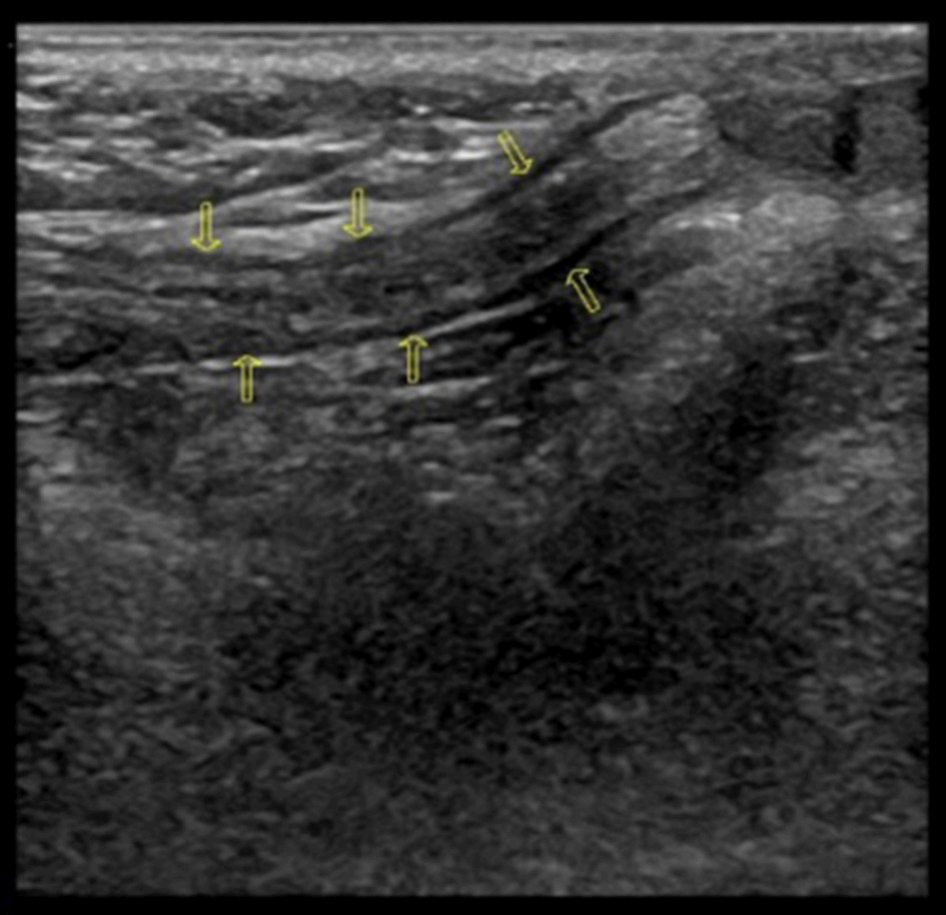
Normal long-axis section of spermatic cord (Yellow arrows point to hypoechoic cremaster muscles).

**Figure 2:**
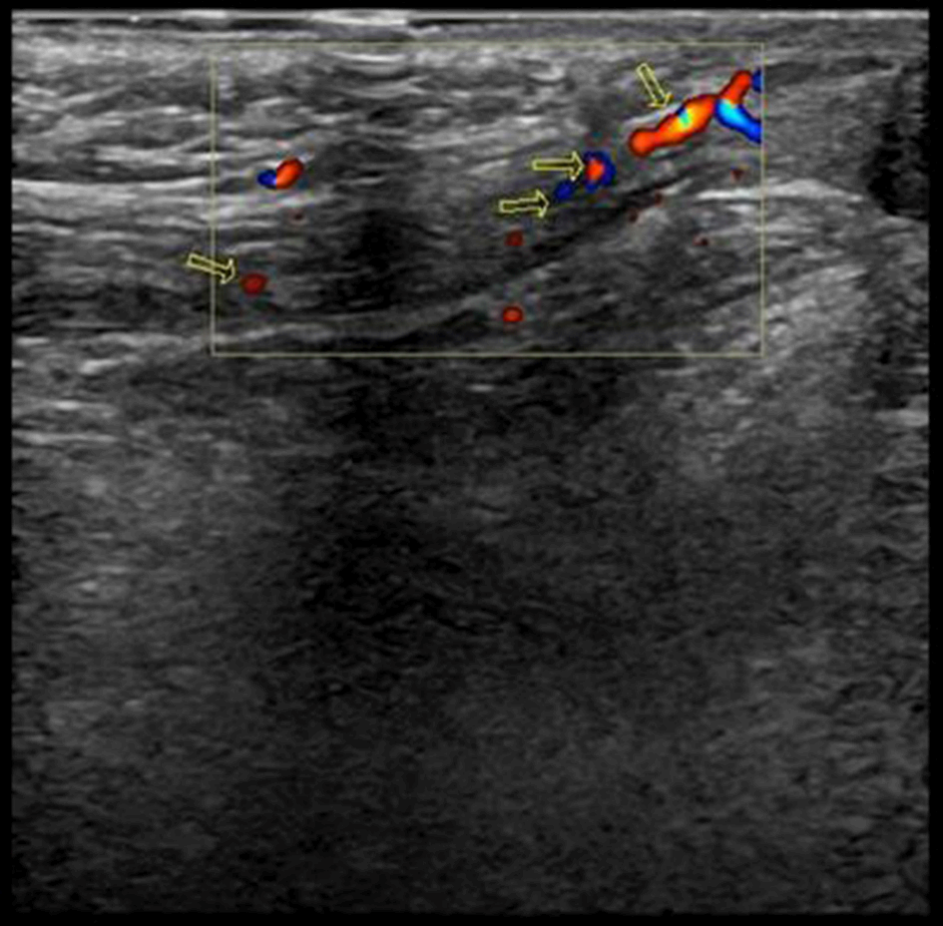
Color Doppler Flow Imaging (CDFI) showed the flow signal.

**Figure 3:**
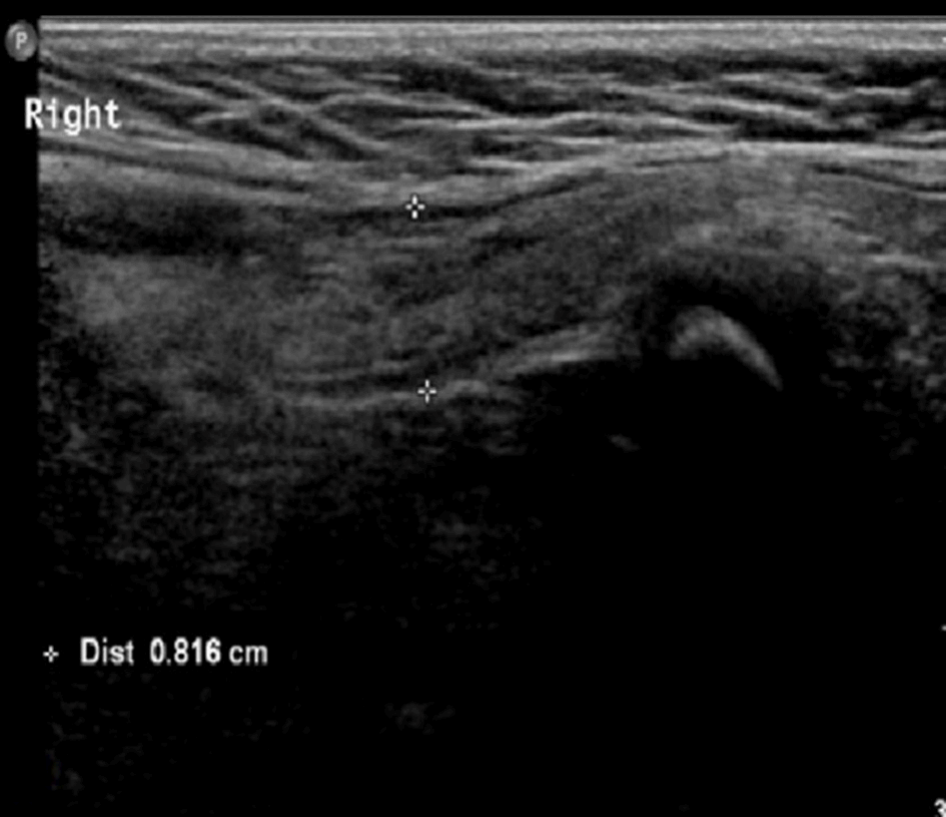
Diameter of the hernia-sided spermatic cord.

**Figure 4:**
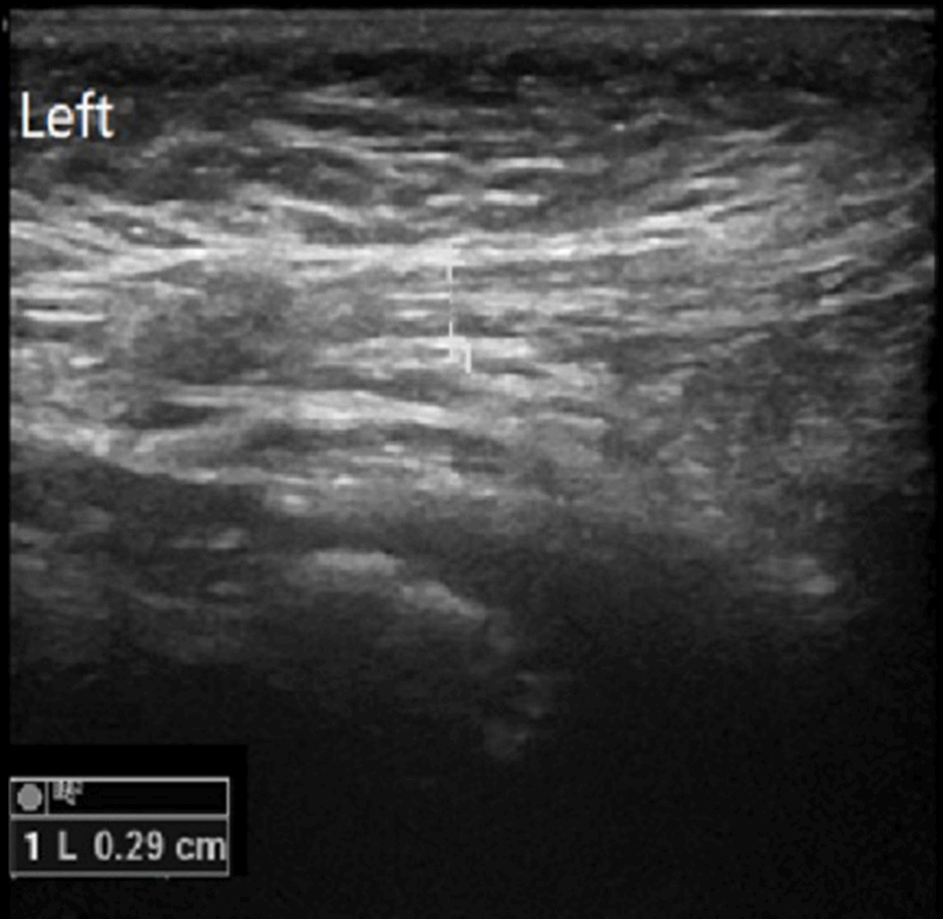
Diameter of the normal side.

Both sides of the spermatic cord were examined by radiologists at the department of ultrasonography with a 7.5-MHz linear transducer; and radiologists were not blind to the clinical diagnosis. Gray-scale ultrasound was performed to show long-axis section of spermatic cord (Figure 1). Then color Doppler Flow Imaging (CDFI) was performed to show the flow signal with the scale adjusted to 2 mm/s (Figure 2). The cremaster muscles were clearly shown (hypoechoic by gray-scale ultrasound) (Figure 1, yellow arrow). In the thickest place, we measured the diameter between the outer edges of cremaster muscles (Figures 3, 4). The maximum diameter of cord was recorded.

### Open herniorrhaphy

Procedures were performed under caudal anesthesia. The inguinal hernia sac was ligated on the symptomatic side without any intervention on the contralateral side unless the sonographic width of the asymptomatic-sided spermatic cord was ≥0.5 cm, and if the sac was identified and opened near the external ring, and CIH was confirmed. Briefly, a small transverse dermatoglyphic incision was made on the symptomatic side, and then Scarpa's fascia was spread. The external oblique was kept intact and the cord was identified near the external ring. After the sac was identified and opened, the cord and vessels were bluntly separated with gauze toward the neck of the sac. Then, the sac was doubly ligated, and the incision was closed with one single suture.

### Follow-up protocol

Follow-up assessments were completed via phone inquiry. The only question was whether the boy developed MIH within 2 years after the initial herniorrhaphy.

### Statistical analysis

Age at initial operation, weight, initial operation side, the sonographic width of spermatic cord, CIH and MIH were collected. Continuous data are expressed as the mean ± SD and were analyzed using two-sample *t*-tests. Comparisons of width of normal side spermatic cord relative to age and weight were analyzed and compared using Spearman's rank correlation coefficient (Spearman's rho). χ^2^ tests were used to determine the significance of differences in the incidence of MIH between the US group and the non-US group. A receiver operator curve (ROC) analysis was performed to evaluated predictive value of the sonographic width of the spermatic cord for contralateral hernia. *P* < 0.05 was considered statistically significant. Statistical analyses were performed with SPSS.

## Results

A total of 2978 of all patients were boys aged 1–2 years (13–24 months) with a unilateral inguinal hernia; 24-month follow-up data were obtained from 1,793 (60.2%) boys. There were 1,162 participants in the US group (408 on the left side, 754 on the right side) and 631 in the non-US group (196 on the left side, 435 on the right side). Of the 1,162 boys in the US group, the sonographic width of the hernia-sided spermatic cord (0.75 ± 0.18 cm) was larger than the normal side (0.37 ± 0.05 cm, *P* < 0.001, see Table 1). And the width of normal side spermatic cord had no significant difference between the groups regarding the factors such as age and weight (Table 2). There were 66 boys (46 with an initial left-sided inguinal hernia, 20 with an initial right-sided inguinal hernia) in the US group in whom the width of the asymptomatic-sided spermatic cord was ≥0.5 cm, and these patients underwent contralateral groin exploration. Of the 66 boys, CIH was confirmed in 57 boys: 41 on the right side and 16 on the left side. Unfortunately, 9 boys exhibited negative findings. Among the 66 boys, 18 boys in whom the width of the asymptomatic-sided spermatic cord was ≥0.55 cm were all confirmed CIH. No inguinal hernia or MIH was reported when the width of the normal-sided spermatic cord was ≤ 0.35 cm. MIH developed in 74 boys (74/1793, 4.1%), see Table 3. The incidence of MIH in the US group was 2.2% (25/1162) much lower than 7.8% (49/631) in the non-US group (*P* < 0.001) due to the simultaneous operation of the contralateral side during the first operation in whom the width of the asymptomatic-sided spermatic cord was ≥0.5 cm. Among the MIH boys, there were 25 boys in the US group (right-sided MIH 8/25, 32%; left-sided MIH 17/25, 68%) and 49 in the non-US group (right-sided MIH 38/49, 77.6%; left-sided MIH 11/49, 22.4%). Among the 25 MIH boys in the US group, generally the sonographic width of the normal-sided spermatic cord was between 0.4 and 0.45 cm; only 2 (4%) boys had widths of less than 0.4 cm (0.37 and 0.36 cm, respectively). The width of the spermatic cord predicted the presence of contralateral hernia with ROC area under the curve was 0.943 (95% CI = 0.919–0.966). We analyzed the incidence of contralateral hernia using width of the spermatic cord at every point count for the 1,162 patients. If the width of the asymptomatic-sided spermatic cord was 0.5 and 0.54 cm, the corresponding sensitivity was 0.682 and 0.294, respectively, and the corresponding specificity was 0.991 and 1.000, respectively. The cost of spermatic cord ultrasonography was 10 USD/patient, and the cost of extra operative sessions of MIH was 1420 USD/patient. In this study we detected 57 CIH, and we proposed to eliminate 57 MIH. So the financial cost of pre-operative us examination of the 1,162 patients (11620 USD) was much lower than the cost of extra operative sessions of MIH (80940 USD).

**Table 1:** The sonographic width of spermatic cords.

	**Width of spermatic cord (cm)**		***P*-value**
	**Hernia side**	**Normal side**	
All boys (*n* = 1,162)	0.75 ± 0.18	0.37 ± 0.05	< 0.001
Right-sided inguinal hernia (*n* = 705)	0.76 ± 0.18	0.37 ± 0.05	< 0.001
Left-sided inguinal hernia (*n* = 390)	0.74 ± 0.17	0.36 ± 0.06	0.001

*Values are mean ± SD. independent-samples t-test (width of hernia-sided spermatic cord compared to that of the normal side)*.

**Table 2:** Distribution of weight and width of normal side spermatic cord to age groups.

**Months**	**Cases**	Weight (kg)^*^				**Width of normal side spermatic cord (cm)**^#^			
		**Mean ±*SD***	**Maximum**	**Median**	**Minimum**	**Mean ±*SD***	**Maximum**	**Median**	**Minimum**
13	148	10.5 ± 0.9	12.5	10.5	8.6	0.37 ± 0.06	0.56	0.36	0.23
14	185	10.6 ± 0.9	12.9	10.5	8.9	0.37 ± 0.06	0.57	0.37	0.28
15	159	10.7 ± 0.9	13	10.6	9.1	0.38 ± 0.06	0.55	0.38	0.25
16	111	10.9 ± 0.9	13.1	10.8	9.4	0.38 ± 0.06	0.58	0.37	0.27
17	125	11.0 ± 1.0	13.2	11	9.5	0.37 ± 0.07	0.56	0.36	0.26
18	98	11.3 ± 0.9	13.4	11.2	9.6	0.38 ± 0.07	0.54	0.38	0.25
19	72	11.3 ± 0.9	13.6	11.1	9.7	0.39 ± 0.06	0.56	0.38	0.28
20	70	11.3 ± 0.9	13.3	11.3	9.9	0.37 ± 0.06	0.54	0.37	0.29
21	56	11.5 ± 1.0	13.5	11.4	9.9	0.38 ± 0.08	0.58	0.38	0.24
22	36	11.7 ± 0.9	14	11.6	10.3	0.40 ± 0.05	0.52	0.4	0.31
23	51	11.7 ± 1.0	13.8	11.6	10.1	0.37 ± 0.06	0.57	0.37	0.25
24	51	11.9 ± 1.0	14.3	11.6	10.5	0.39 ± 0.08	0.57	0.4	0.23

*Width of normal side spermatic cord relative to age and weight were compaired using Spearman's rho*.

* correlation coefficient = 0.034, P = 0.241;

# *correlation coefficient = 0.042, P = 0.156*.

**Table 3:** Cases of MIH based on the laterality of the initial inguinal hernia.

**Number of MIH cases**	**US group (*n* = 1162)**	**non-US group (*n* = 631)**
Right-sided MIH*	8	38
Left-sided MIH*	17	11
Total cases	25^&^ (2.2%)	49^&^ (7.8%)

MIH, metachronous inguinal hernia. *χ^2^ test (the laterality of MIH between the US and non-US groups), P < 0.001; ^&^χ^2^ test (the total incidence of MIH between the US and non-US groups), P < 0.001.

## Discussion

According to our previous study, MIH developed mostly in boys under 2 years old and within 2 years after the initial herniorrhaphy (2). Therefore, this study, we focused on boys under 2 years old and set the follow-up period to 2 years. We found that 1. The sonographic width of the hernia-sided spermatic cord was larger than the normal side. 2. The width of normal side spermatic cord had no significant difference between the groups regarding the factors such as age and weight. 3. The incidence of MIH in the US group was 2.2% much lower than 7.8% in the non-US group due to the simultaneous operation of the contralateral side during the first operation in whom the width of the asymptomatic-sided spermatic cord was ≥0.5 cm. 4. The width of the spermatic cord predicted the presence of contralateral hernia with ROC area under the curve = 0.943 (95% CI = 0.919–0.966).

Herniorrhaphy is the most common procedure in a children's hospital. MIH is a concern of surgeons as well as patients. Surgeons always make efforts to find ways to accurately assess a true CIH to avoid unnecessary exploration, remove the need for a second operation and reduce costs. However, no methods have been sufficiently popular to be widely introduced into daily practice.

LHR can confirm the presence of CPPV. However, the incidence of CPPV evaluated by laparoscopy is 20–51.5%, which is much higher than the natural incidence of CIH or MIH. Laparoscopy allows for easy evaluation of CPPV, but the clinical significance of and need for repair of an identified defect is unclear and laparoscopy increase the risk of iatrogenic ascending testis (9–11). Furthermore, the risk of developing an asymptomatic hernia during childhood in the presence of a known CPPV is relatively low, and the rate of CPPV decreases with increasing age(4, 12). Therefore laparoscopy is a sensitive method with which to detect CPPV and a good way to prevent MIH at the expense of an overtreatment of CPPV, but it is not a precise way to predict CIH. Importantly negative evaluation of CPPV does not exclude the possibility of CIH (13).

The odds of having MIH were significantly higher in children with an initial left-sided hernia and in children with CPPV (14, 15). In the non-US group, 77.6% of patients developed MIH following initial left-sided herniorrhaphy. However, in the US group, the results were reversed: 68% of patients developed MIH following an initial right-sided herniorrhaphy. What factors are responsible for the change? According to pre-operative spermatic cord ultrasonography, we detected 57 CIHs (41 on right side and 16 on left side) and the incidence of MIH decreased from 7.8% in the non-US group to 2.2% in the US group due to the simultaneous operation of the contralateral side during the first operation in whom the width of the asymptomatic-sided spermatic cord was ≥0.5 cm. Therefore, under the guide of pre-operative spermatic cord ultrasonography, we reduced the development of MIH, especially right-sided MIH.

To evaluate the width of the spermatic cord, ultrasonography is a safe, non-invasive and repeatable method (6, 8, 16, 17). Thickening of the spermatic cord is a clinical sign of an inguinal hernia. And our study showed that the width of normal side spermatic cord had no significant difference between the groups regarding the factors such as age and weight. So we raised the hypothesis that the sonographic width of spermatic cord could predict the presence of inguinal hernia. The width of the spermatic cord predicted the presence of contralateral hernia with ROC area under the curve = 0.943 (95% CI = 0.919–0.966). If the width of the asymptomatic-sided spermatic cord was 0.5 cm and 0.54 cm, the corresponding sensitivity was 0.682 and 0.294, respectively, the corresponding specificity was 0.991 and 1.000, respectively. This result hinted that the sonographic width of spermatic cord was a fine indicator to predict the presence of inguinal hernia. Actually, of the 66 boys in whom the width of the spermatic cord on the asymptomatic side was ≥0.5 cm, 57 (86.4%) boys were confirmed as having CIH, and only(13.6%) boys received negative findings. 18 out of the 66 boys in whom the width of the asymptomatic-sided spermatic cord was ≥0.55 cm, all had confirmed CIH. No inguinal hernia was present when the width of the normal-sided spermatic cord was ≤ 0.35 cm. So if the width of spermatic cord was ≥0.5 cm, it dramatically hinted CIH, if it was ≥0.55 cm, CIH was confirmed, and if it was ≤ 0.35 cm, it excluded CIH.

As we calculated that the financial cost of pre-operative us examination of the 1,162 patients (11620 USD) was much lower than the cost of extra operative sessions of MIH (80940 USD). And the incidence of MIH was low in US group. So our study would achieve the aim to remove the need for a second operation and reduce costs.

Therefore, in short, if the width of the asymptomatic-sided spermatic cord of boys with initial unilateral inguinal hernia was ≥0.5 cm, contralateral groin exploration was recommended, and it help to reduce the incidence of MIH.

## Author contributions

SH and ZZ: study conception and design; CL, XY, YQ, and SH: data acquisition; JL: analysis and data interpretation; SH: drafting of the manuscript; SH and ZZ: critical revision.

### Conflict of interest statement

The authors declare that the research was conducted in the absence of any commercial or financial relationships that could be construed as a potential conflict of interest.
